# A Unique Case of Rapidly Progressive Glomerulonephritis in a Patient With Anti-neutrophil Cytoplasmic Antibody (ANCA)-Positive Vasculitis Presenting With Ocular and Cardiac Manifestations

**DOI:** 10.7759/cureus.77100

**Published:** 2025-01-07

**Authors:** Sylvia Li, Rachel Sergah, Ivan Roubal, Kyle Chang, Dan Vo

**Affiliations:** 1 Internal Medicine, Arrowhead Regional Medical Center, Colton, USA; 2 Internal Medicine, California University of Science and Medicine, Colton, USA

**Keywords:** anca-associated vasculitis, anti-neutrophil cytoplasmic antibody (anca), autoimmune vasculitis, granulomatosis with polyangiitis (gpa), pr3-anca, rapidly progressive glomerulonephritis (rpgn), rpgn, wegener’s granulomatosis

## Abstract

Anti-neutrophil cytoplasmic antibody (ANCA)-associated vasculitides (AAVs) can be divided into three distinct clinical entities. Of the three subgroups, granulomatosis with polyangiitis (GPA) is the most common AAV. We present a 33-year-old Hispanic male with no past medical history who presented to the ER with acute-onset pleuritic chest pain and dyspnea. The patient had chronic sinusitis with occasional epistaxis. Initial lab work was significant for hematuria and proteinuria, as well as worsening acute kidney injury (AKI). A renal biopsy confirmed the presence of rapidly progressive glomerulonephritis (RPGN). Autoimmune panels were significant for PR3-ANCA positivity. The patient’s clinical picture was most compatible with that of GPA, given the prominence of upper airway symptoms in the setting of new-onset renal failure with erythrocyturia and proteinuria, as well as pulmonary lesions. Cyclophosphamide-based treatment was initiated, in conjunction with glucocorticoids, mesna, and atovaquone. High suspicion for GPA with RPGN should be considered in the younger population who present with new-onset renal failure and pulmonary lesions to present irreversible kidney injury.

## Introduction

The anti-neutrophil cytoplasmic antibody (ANCA)-associated vasculitides (AAVs) are a collection of relatively rare autoimmune diseases predominantly affecting small vessels and are characterized by autoantibodies targeting neutrophil proteins leukocyte proteinase 3 (PR3-ANCA) or myeloperoxidase (MPO-ANCA). These AAV diseases can be divided into three distinct clinical entities including granulomatosis with polyangiitis (GPA), eosinophilic GPA, and microscopic polyangiitis, which may be differentiated from one another by clinical features, type of organ involvement, histopathologic evidence of granulomatous vasculitis, and ANCA serotype [1].

Of the three subgroups, GPA is the most common AAV with an incidence of five to 10 cases per million depending upon the geographical area, with a greater association in higher latitudes. GPA is more commonly documented in Caucasians and is generally seen in the geriatric population with a peak at 64-75 years of age. Nonetheless, it may still be present in younger individuals and all racial groups, thus posing a great diagnostic challenge as it was in our clinical case [2,3].

Clinically, GPA comprises a triad of necrotizing granulomas of upper and lower respiratory tracts, systemic vasculitis, and necrotizing glomerulonephritis. Multisystem involvement and varied presentations with non-specific symptoms, which may overlap with or mimic other more common conditions, furthermore make GPA a difficult yet intriguing diagnosis. As with other AAV diseases, early diagnosis and management are critical to prevent the development of rapidly progressive glomerulonephritis (RPGN), a severe and potentially life-threatening complication characterized by a rapid decline in kidney function over days to weeks.

In this article, we present an interesting case of GPA with PR3-ANCA-positive RPGN in a young Hispanic, non-Caucasian male. We also explore the differential diagnosis relative to other potential AAV causes of RPGN and discuss its treatment.

## Case presentation

A 33-year-old Hispanic male with no significant past medical history presented to the ER in January 2024 with worsening pleuritic chest pain and dyspnea for the past three days. The patient reported that he was recently seen for sinusitis in November 2023 and had subsequently completed a seven-day course of amoxicillin-clavulanate. During that time, the patient noted that he had experienced bloody and mucinous nasal discharge through the end of December, which has since resolved. The patient also reported difficulty ambulating due to dyspnea on exertion and generalized weakness. Since November, he has had 26 pounds of unintentional weight loss with night sweats. Finally, he also reported one month of redness in both eyes accompanied by floaters. The patient denied any discharge from the eyes, blurry vision, and eye pain.

**Figure 1:**
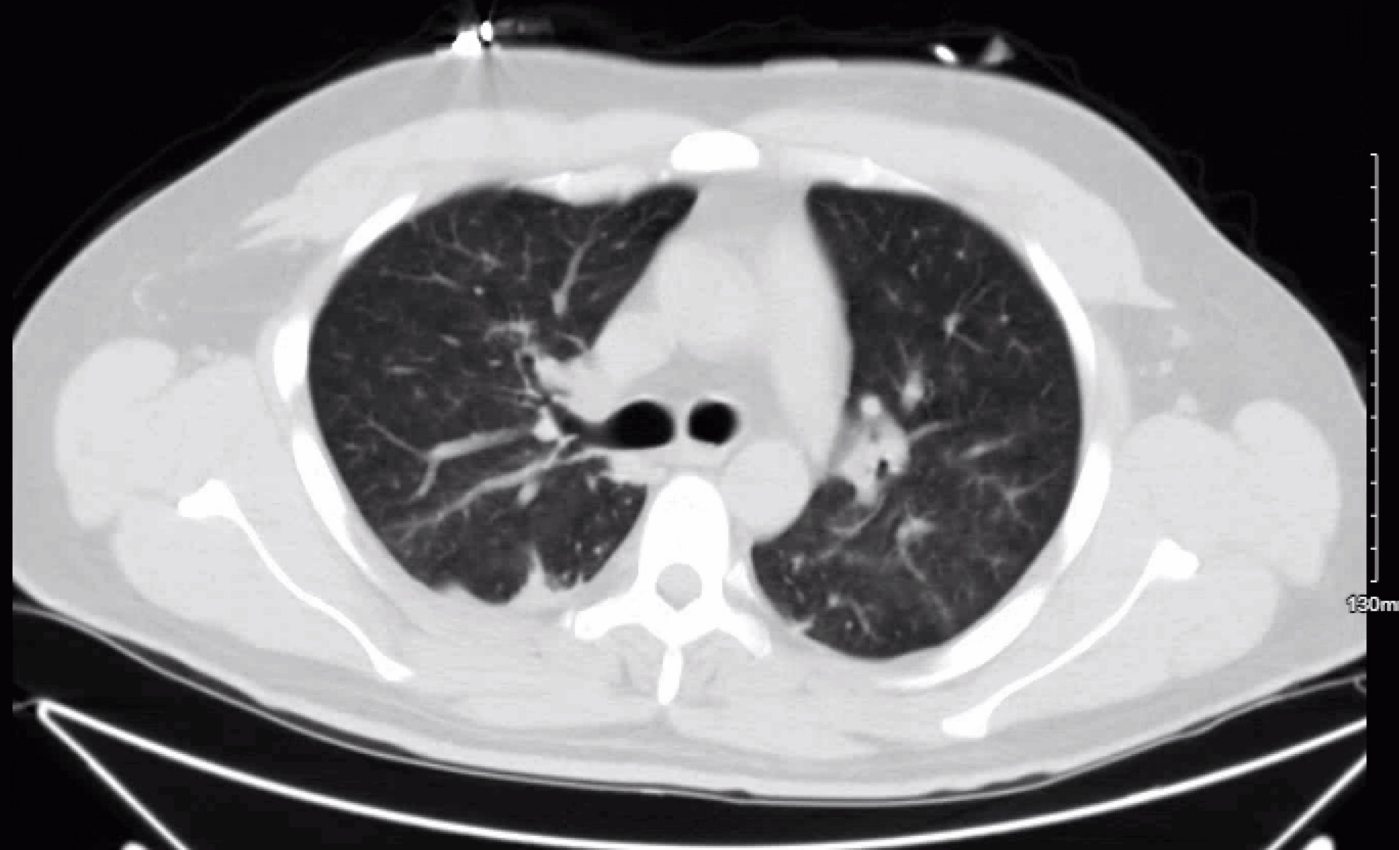
CT chest without contrast during the first hospitalization

At the time he presented to our ER, he was febrile with malaise and shortness of breath. He otherwise denied having nausea, vomiting, diarrhea, skin changes, edema, dysuria, sick contacts, or any other additional symptoms. Vitals were significant for heart rate of 122 beats per minute, respiratory rate of 19 breaths per minute, blood pressure of 128/82 mmHg, and oxygen saturation of 94% on room air. On physical exam, the patient was noted to have injected sclera without purulent discharge. No midline defects of the nasal bridge or septum were noted; the patient had tenderness to percussion of the right anterior cheek below the inferior orbital margin. Lung sounds were clear to auscultation bilaterally with no wheezes, rhonchi, or rales. Heart sounds revealed a regular rate and rhythm, with no murmurs. Laboratory workup (Table 1) was notable for a white blood cell count of 17,100/uL, microcytic anemia with hemoglobin of 12.2 g/dl, mild hyponatremia of 129 mmol/L, blood urea nitrogen of 54, creatinine of 4.98, with negative troponins, negative respiratory Biofire panel, and a B-type natriuretic peptide (BNP) within normal limits. Urinalysis revealed 2+ protein, 3+ blood, and 30-60 pRBCs, with no casts seen. Renal ultrasound demonstrated echogenic kidneys suggestive of medical renal disease without evidence of obstructive uropathy, and CT chest without contrast (Figure 1) revealed mild infiltrate on the left lung base, subsegmental atelectasis, and trace right pleural effusion. The patient was given 30 ml/kg of NS and a 2 g dose of ceftriaxone before subsequent hospital admission for acute renal failure and meeting systemic inflammatory response syndrome criteria.

**Table 1:** Laboratory values on the first admission WBC: white blood cell; Hgb: hemoglobin; MCV: mean corpuscular volume; BUN: blood urea nitrogen; eGFR: estimated glomerular filtration rate

Laboratory study	Lab values	Reference range
WBC	17.1 10*3/uL	4.5-11.1 10*3/uL
Hgb	12.2 g/dL	13.0-17.0 g/dL
MCV	74 fL	80-100 fL
Neutrophils relative	77%	38-75%
Platelets	472 10*3/uL	120-360 10*3/uL
Sodium	129 mmol/L	135-148 mmol/L
BUN	54 mg/dL	8-20 mg/dL
Creatinine	4.98 mg/dL	0.50-1.50 mg/dL
eGFR	14.8 L/min/1.73m*2	≥90 L/min/1.73m*2
Glucose	128 mg/dL	65-125 mg/dL
Color, urine	Yellow	Dark Yellow, Green, Other, Straw, Yellow
Clarity, urine	Cloudy	Clear
Protein, urine	2	Negative
Blood, urine	3	Negative
Protein, total, random urine	97 mg/dL	5-25 mg/dL

**Figure 2:**
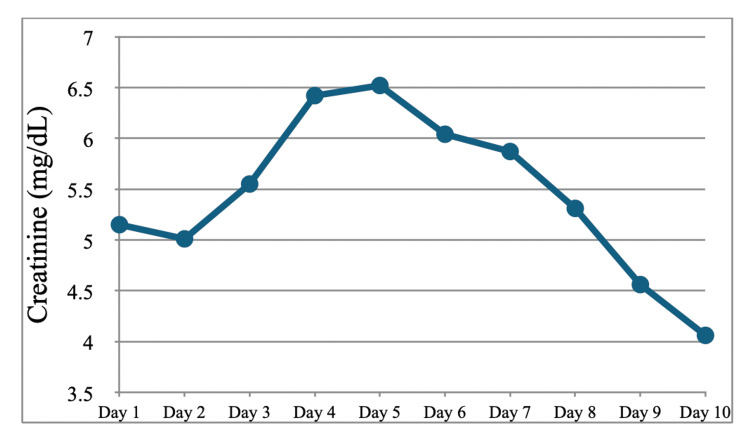
Serum creatinine during the first hospitalization

Ceftriaxone 2 g IV every 24 hours and azithromycin 500 mg IV every 24 hours were empirically started to cover community-acquired pneumonia (CAP), and autoimmune panels were ordered after ESR and CRP levels were found to be markedly elevated. Pulmonology and Nephrology were consulted. After five days of CAP coverage, IV ceftriaxone and azithromycin were discontinued. No more antibiotics were continued in the absence of signs of infection; blood cultures drawn on the day of admission showed no growth at five days, and the patient had remained afebrile throughout the hospital course thus far. Renal biopsy was also recommended by Nephrology, and the procedure was performed by IR on hospitalization day 4. X-ray of the paranasal sinuses demonstrated air-fluid levels in the right maxillary sinus consistent with sinus disease. Renal function also deteriorated during this time, with creatinine increasing from 5.15 to 6.42. Protein-creatinine ratio of the urine resulted in 1,813 mg/g (reference range 25-148 mg/g). Methylprednisolone 500 mg IV daily was started on day 4 of hospitalization after a renal biopsy had been obtained. Per Nephrology, cyclophosphamide therapy 7.5 mg/kg (for GFR <30) was started preemptively on day 5 of hospitalization before finalized renal biopsy results. In the meantime, autoimmune panels returned positive for PR3-ANCA. Atovaquone was started for pneumocystis jiroveci pneumonia (PJP) prophylaxis, and mesna was started to prevent hemorrhagic cystitis while on cyclophosphamide. After beginning the treatment regimen, the patient’s creatinine began to show improvement, downtrending on day 7 (Figure 2).

On day 8 of hospitalization, the patient became bradycardic with a heart rate in the 40s. The patient’s EKG showed sinus bradycardia with first-degree atrioventricular (AV) block. Of note, the patient’s EKG on admission showed first-degree AV block. Cardiology was consulted for evaluation; the service recommended four weeks of cardiac event monitor on discharge.

For the bilateral eye redness, the patient was evaluated by Ophthalmology. Physical exam showed no signs of disc edema, vitritis, or chorioretinal lesions. 2+ inflammation of the sclera was noted bilaterally, blanchable with phenylephrine injections. Ophthalmology agreed with the diagnosis of uveitis and recommended cyclopentolate and prednisone eye drops. After initiating the eye drops, the patient's uveitis began to improve. Also recommended were tests listed in Table 2.

**Table 2:** Serum laboratory studies recommended by Ophthalmology PCR: polymerase chain reaction

Laboratory study	Lab values	Reference range
Lyme disease (Borrelia spp) DNA, qualitative real-time PCR, blood	Not detected	Not detected
Angiotensin-converting enzyme	27 U/l	9-67 U/L
Lysozyme (muramidase)	20.4 mcg/mL	5.0-11.0 mcg/mL
HLA-B27 antigen	Negative	Negative
Quantiferon TB gold plus	Negative	Negative

**Figure 3:**
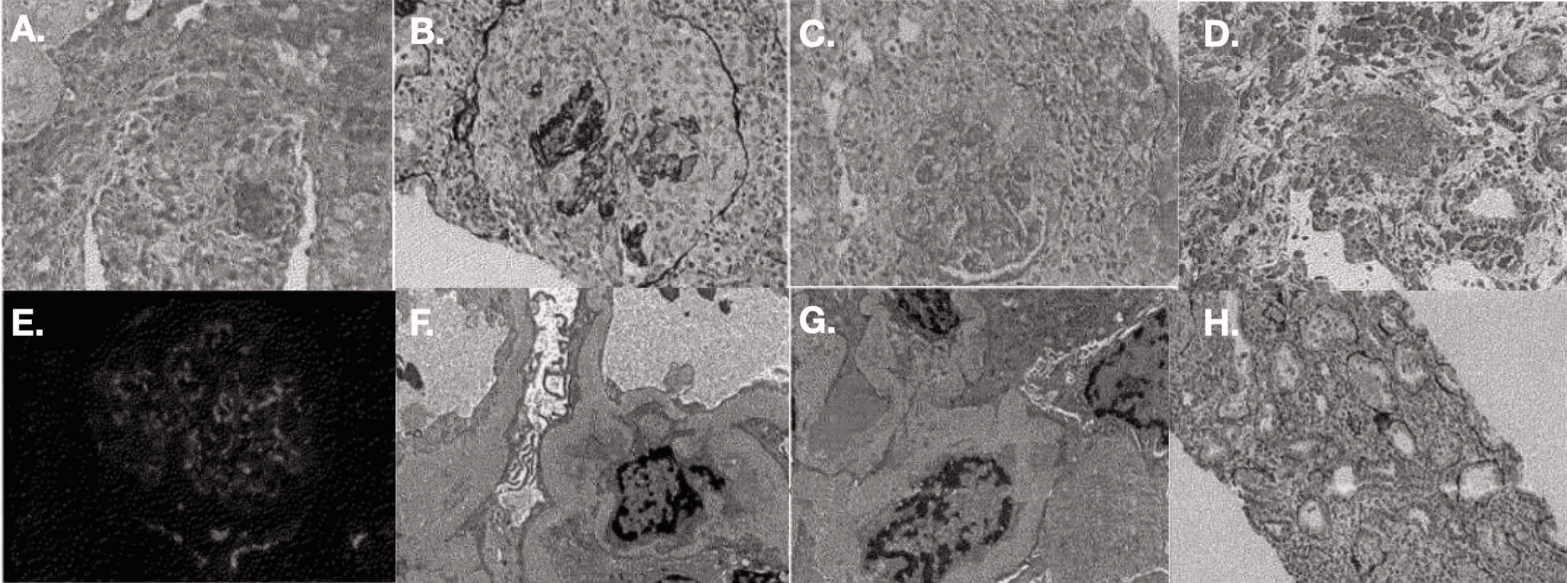
Renal biopsy with light, immunofluorescence, and electron microscopy specimens A. fibrinoid necrosis; B. cellular crescent; C. fibrocellular crescent; D. necrotizing arteritis; E. glomerular C3; F. mesangial deposits; G. subepithelial deposits; H. acute tubulointerstitial nephritis (ATN)

Renal biopsy resulted in findings consistent with diffuse active necrotizing and crescentic glomerulonephritis, as well as the following findings: segmentally thickened glomerular capillary basement membranes, acute tubulointerstitial nephritis, acute tubular necrosis, and mild arteriosclerosis (Figure 3). Moreover, 75% acute and 3% subacute crescents and focal necrotizing arteritis were noted, consistent with the picture of diffuse active RPGN. Also consistent with RPGN were acute tubulointerstitial nephritis and acute tubular necrosis. Segmentally thickened glomerular capillary basement membranes were consistent with prediabetes.

**Figure 4:**
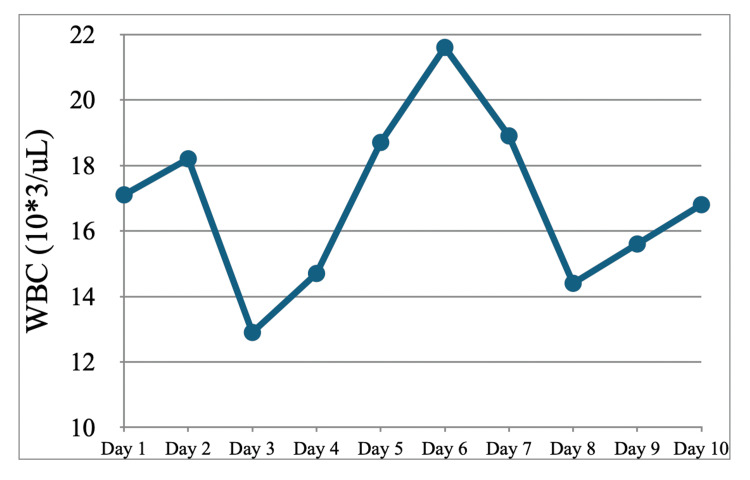
Serum white blood cell count during the first hospitalization WBC: white blood cell count

As mentioned previously, the patient's blood cultures were negative for growth at five days. A transthoracic echocardiogram (TTE) was done, showing no signs of vegetation, after which cyclophosphamide was started. A confirmatory transesophageal echocardiogram (TEE) was completed prior to discharge, also negative for vegetation. ID specialist was consulted, who agreed that there was low suspicion for active infection and that no antibiotics were needed at the time; they recommended several tests to rule out other potential sources of infection, which were ordered (Table 3). The patient remained afebrile throughout the rest of the hospital course after initial presentation to the ED, although leukocytosis with neutrophilic predominance persisted throughout the hospital stay (Figure 4).

**Table 3:** Laboratory studies recommended by infectious diseases PCR: polymerase chain reaction; EIA: enzyme immunoassay; IgG: immunoglobulin G; IgM: immunoglobulin M; VCA: viral capsid antigen; EBNA: Ebstein-Barr virus nuclear antigen

Laboratory study	Lab Values	Reference Range
Parvovirus B19 DNA, qualitative real-time PCR	Not detected	Not detected
*Brucella* antibodies (IgG) EIA	0.49	<0.80
*Brucella* antibodies (IgM) EIA	0.12	<0.80
*Coccidioides* antibody, complement fixation, serum	<1:2	<1:2
*Bartonella henselae *antibodies (IgG)	Negative	Negative
*Bartonella henselae* antibodies (IgM)	Negative	Negative
*Bartonella quintana *antibodies (IgG)	Negative	Negative
Bartonella quintana antibodies (IgM)	Negative	Negative
Q fever (*Coxiella burnetii*) phase I antibodies (IgG)	Negative	Negative
Q fever (*Coxiella burnetii*) phase II antibodies (IgG)	Negative	Negative
Q fever (*Coxiella burnetii*) phase I antibodies (IgM)	Negative	Negative
Q fever (*Coxiella burnetii*) phase II antibodies (IgM)	Negative	Negative
Epstein-Barr virus VCA Ab (IgM)	<36.00 U/mL	<36.00 U/mL
Epstein-Barr virus VCA Ab (IgG)	>750.00 U/mL	<18.00 U/mL
Epstein-Barr virus EBNA Ab (IgG)	>600.00 U/mL	<18.00 U/mL
Cytomegalovirus antibody (IgG)	6.90 U/mL	<0.60 U/mL
Cytomegalovirus antibody (IgM)	<30.00 AU/mL	<30.00 AU/mL
Cytomegalovirus DNA, quantitative, real-time PCR	Not detected	Not detected

With ANCA vasculitis confirmed, the patient was eventually tapered from methylprednisolone to prednisone 80 mg. Once the patient’s creatinine had demonstrated a consistent downward trend for three days, on day 10, the patient was discharged with the following regimen: he was instructed to continue cyclophosphamide every two weeks with two doses, then every three weeks for six months with mesna and atovaquone, with arrangement for outpatient Nephrology follow-up. The patient was seen in the Nephrology Clinic within one week of discharge. There, avacopan 30 mg twice daily was started; prednisone dose was reduced to 60 mg daily from 80 mg daily; cyclophosphamide and mesna were continued, scheduled monthly for six months; atovaquone was continued as well. The patient received cyclophosphamide and mesna infusions for one visit before returning to the ED one month after the first hospital discharge.

**Figure 5:**
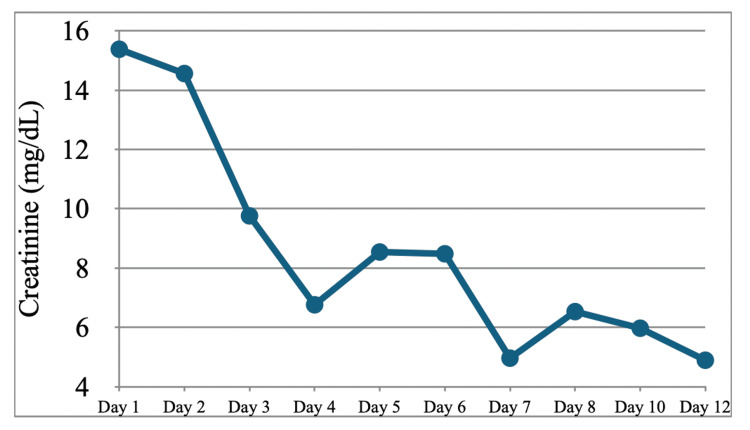
Serum creatinine during the second hospitalization

The patient presented again to the ED complaining of one week of emesis and malaise, interfering with his ability to take his medications. Chart review revealed that the anti-GBM antibody, which had been pending on discharge from the last hospitalization, was negative. Labs on this admission were significant for anemia with hemoglobin of 7.6, bicarbonate of 18, BUN of 131, and creatinine of 15.38. Urinalysis was significant for WBC 5-20, RBC 30-60, with granular and hyaline casts. The urine albumin-to-creatinine ratio was 2012.3. Nephrology was consulted promptly while the patient was in the emergency department, and on hospital day 3, tunneled dialysis catheter was placed, with hemodialysis starting the same day. His creatinine downtrended with consistent hemodialysis (Figure 5), and creatinine on discharge was 4.98. The patient was subsequently lost to follow-up, with future appointments cancelled.

## Discussion

GPA is an autoimmune vasculitis affecting multiple organ systems. It is accompanied by renal involvement in up to 83% of cases [1]. RPGN is renal failure characterized by glomerulonephritis, with findings of red blood cells and protein in the urine. RPGN can be further subcategorized, one category being pauci-immune glomerulonephritis. PICG is often found presenting in conjunction with small vessel vasculitides such as GPA. Of clinical importance, without treatment, PICG has a one-year mortality of 80% [4,5]. Of those who receive treatment, 75% at five years survive. However, there is a high rate of relapsing RPGN, up to 40%, and up to 25% of patients progress to end-stage renal disease [6].

c-ANCA is positive in 90% of patients with active GPA and may have implications in clinical presentation and course [7]. Testing positive for c-ANCA is associated with a higher relapse rate for glomerulonephritis and renal failure, and it has been speculated that there is a potential role of causation of c-ANCA as a trigger for GPA [4]. Destructive lesions in the nasal septum have 90% c-ANCA positivity. Lung nodules and cavities more often c-ANCA, as opposed to pulmonary capillaritis and the distinct lack of nodules or cavities for p-ANCA-positive patients. On histology, GPA is characterized by the classic triad of vasculitis, necrosis, and granulomatous inflammation in 16% of the cases, usually from tissue samples of lung lesions [4]. The renal biopsy findings of diffuse active necrotizing and crescentic glomerulonephritis can also be found in infection-related glomerulonephritis, specifically endocarditis-associated glomerulonephritis [8]. An extensive workup was performed on our patient to rule out infection and infectious endocarditis (IE); the workup for the latter included blood cultures, TTE, and TEE, which were negative for signs of IE. Our patient did have persistent leukocytosis during the first hospital stay in the absence of any sign of active infection. However, in GPA, it is common for patients to have leukocytosis with neutrophilic predominance, without an infectious etiology present [9]. We began corticosteroids and cyclophosphamide early on in the patient's course, as infection and IE were very low on our differential. However, it is important to keep in mind that should a patient with IE-associated glomerulonephritis be treated with immunosuppressants, mortality rates are as high as 11% despite improvements in renal function [10].

Treatment of GPA, as in our patient, usually consists of cyclophosphamide in conjunction with systemic glucocorticoids, although other combinations of immunosuppressants can be used. The remission rate for those treated with the aforementioned regimen is 75% at three months and 90% at six months [6]. The treatment regimen, as in our patient, usually includes mesna for prevention of cyclophosphamide-induced hemorrhagic cystitis, as well as atovaquone for PJP prophylaxis, as treatment regimen of glucocorticoids and cyclophosphamide have strong immunosuppressive effects. Plasma exchange was not considered for our patient, as the role of plasma exchange in severe AAV is controversial. In the 2007 MEPEX trial (n = 137), plasma exchange was associated with a decreased risk of developing ESRD [11]. However, the 2020 PEXIVAS trial (n = 704) showed no relation between the use of plasma exchange and the risk of ESRD in patients with severe AAV, although there may be some benefit for those with diffuse alveolar hemorrhage {DAH) [12]. Our patient had none of the characteristic ground-glass opacities or centrilobular nodules on the CT chest, nor did he present with hemoptysis or cough. However, our patient did present with fever and chest pain. The patient did have a trace right-sided pleural effusion, which may also be the culprit of his chest pain, especially considering the pleuritic characterization of his pain.

In our case, both GPA and MPA were high on our differential. However, the patient’s lack of neuropathy, p-ANCA, and constitutional symptoms such as weight loss or fever, lower MPA on our differential [8]. Evidence of sinusitis and upper airway involvement in addition to the presence of positive c-ANCA are more strongly supportive of GPA as the clinical picture. Additional diagnoses that had been considered but were much lower on the differential were EGPA, which would have shown eosinophilia in addition to asthmatic symptoms (often progressive and steroid dependent, preceding vasculitis by three to nine years), as well as post-streptococcal glomerulonephritis, which was lower on our differential considering patient had not presented with recent prior upper respiratory tract infection.

Ocular manifestations can occur in up to 60% of patients with GPA, with the frequency of bilateral involvement up to 58% [13]. Every structure of the eye can be affected by GPA [13]. Our patient's presentation of bilateral redness of the eye with floaters was concerning for anterior uveitis. Our patient did not have any discharge from his eyes, lowering conjunctivitis on the differential; in addition, the eye redness had been present for one month without self-resolution also makes conjunctivitis less likely. Uveal involvement in GPA is rare, with frequency reported up to 10%; uveitis usually accompanies a primary other inflammation of the eyes, such as scleritis or episcleritis [14]. Our patient's physical exam having blanching of the sclera upon phenylephrine injection supports that the patient had episcleritis on top of the uveitis. Topical corticosteroids, which were given to our patient, are indicated for treatment of both episcleritis and anterior uveitis; cyclosporine was indicated in our patient due to bilateral ocular involvement [14].

Cardiac manifestations of GPA are rare. The frequency reported in a North American trial by McGeoch et al. was 3.3%, lower than that of the European and French cohorts, which were 5.7% and 13%, respectively [15]. Cardiac conduction abnormalities are rare in the setting of GPA; only 20 cases of high-degree AV block in the setting of GPA have been reported in the medical literature thus far [16]. Granulomatous infiltration of the conduction pathway has been associated with cardiac conduction abnormalities in prior reports [17]. Our patient had had an asymptomatic first-degree AV block that was discovered during hospitalization. Cyclophosphamide-induced AV block was unlikely, as AV block had been present on an EKG on admission prior to starting the medication. The patient is young and reports that he leads an athletic lifestyle, which could be the cause of the conduction abnormality; however, it cannot be excluded that GPA and resulting granulomatous infiltration of cardiac tissue is the cause of his presentation. In the absence of symptoms and a high-degree AV block, our choice of management was continued monitoring without invasive interventions. The association of cardiac involvement in GPA patients with the risk of vasculitis relapse or mortality has conflicting data; the European report supported such an association, while the North American report found no correlation [15].

The main limitation of our study was not having obtained a lung biopsy. CT findings were not positive for characteristic cavitary lung lesions usually seen in GPA, and with a lack of pulmonary lesions, there were no available and appropriate sites to biopsy. The risk of undergoing the procedure outweighed the potential yield of the results. Another limitation of the case is that the renal biopsy result showed histopathological traits not specific to GPA. It is not altogether unusual for biopsy to be negative for granulomas in GPA; only 21% of GPA cases have confirmed granulomas on biopsy [4]. It is our contention that this patient has GPA as opposed to other small-vessel vasculitides causing PICG. Regardless, the patient presented with PICG, for which treatment is uniform across all RPGN associated with AAVs [6].

## Conclusions

A high suspicion for GPA should be maintained when a patient presents with new-onset renal failure with erythrocyturia and proteinuria, as well as pulmonary and/or upper airway lesions. Regardless of whether or not it is confirmed GPA versus MPA versus EGPA - all of which are ANCA-positive - glomerulonephritis in all of them is indistinguishable, although treated the same regardless. Patients who have RPGN in the setting of GPA have a high risk of developing ESRD. Rapid initiation of medical therapy is crucial in patients, such as this one, as the pathological processes of RPGN in GPA have such high rates of mortality. Prompt recognition of RPGN and GPA is crucial for timely intervention. It is important for providers to be aware that, although rare, GPA can have ocular and cardiac involvement. Despite treatment, relapse rates are high. As of now, the data regarding the role of plasmapheresis in preventing progression to ESRD is mixed. Should renal biopsy show diffuse active necrotizing and crescentic glomerulonephritis, IE-associated glomerulonephritis must be considered on the differential; if the infection is high on the differential, caution must be practiced before starting immunosuppressants.
